# Employees and the National Insurance Act

**Published:** 1912-03-16

**Authors:** 


### NATIONAL INSURANCE ACT DEPARTMENT.

### EMPLOYEES AND THE NATIONAL INSURANCE ACT.

### II.—The Constitution of Approved Societies.

Insurance against sickness has in the past been almost exclusively confined to Friendly, Co-operative and Dividing Societies, Trade Unions, and the like. Societies of these descriptions have for many years been granting benefits of a similar character to the principal benefits now conferred by the State scheme, and it was in recognition of this anticipation that the Act was designed to enable existing organisations to administer the benefits. Admirable as had been the work of the Friendly and kindred societies, they had not completely covered the ground, and large numbers of the wage-earning section of the community were left untouched by their operations. In particular very few societies insured women, who were thus left without the inducement to thrift, and in consequence fared badly, or became a charge on the community in the event of prolonged sickness.

#### Compulsory and Former Insurance Systems.

The National Insurance Act has now made insurance compulsory on a section of the community estimated at approximately 13,000,000, or nearly one-third of the entire population, and thus the scope for the societies' operations has been greatly enlarged. Provision has been inserted in the Act which makes it possible for any existing society to take the necessary steps for the formation of a separate section to undertake the State insurance business, notwithstanding anything contrary thereto in its rules or other instrument governing its constitution. Most of these existing societies will, doubtless, take steps to form such separate sections. In some cases the matter has already been taken in hand.

It by no means follows, however, that the existing societies will secure a monopoly of the business. Any society—that is to say, any body of persons having a constitution of such a character that it complies with the requirements of the Act relating to approved societies—may be approved by the Insurance Commissioners. Thus it is open to the large Industrial Assurance Companies, and Collecting Friendly Societies, which in the past have confined their operations in large measure to funeral benefits, to form separate sections for the purposes of the Act. In addition to these there will doubtless be many shop clubs and superannuation funds, with existing organisations, in connection with large institutions—such, for instance, as railway companies and large business houses—which will desire to make use of the facilities afforded by the Act for the formation of approved societies with the object of benefiting their members.

#### The Organisation of an Approved Society.

Before a society can commence the transaction of business under the Act it must formally apply to the Insurance Commissioners for approval, and if approved it shall be an approved society for the purposes of the Act. The principal conditions to be satisfied by a society desiring approval are: (1) It must not be a society carried on for profit, and (2) its constitution must provide to the satisfaction of the Insurance Commissioners for its affairs being subject to the absolute control of the members. It will thus be seen that an approved society must be a mutual benefit society, and no part of any profit that may arise on its transactions may be applied as dividends to shareholders or promoters.

The control of the affairs of the society by the members has been carefully guarded by the Act, and provision is made that if there be honorary members—that is, members who are not insured persons—the rules of the society must exclude the right of voting of such honorary members on all questions and matters arising under the Act.

#### The Position of Each Member.

One very important provision that must be carefully borne in mind is that no person may belong or endeavour to gain admittance to more than one approved society for the purposes of the State insurance at the same time, under pain of heavy penalties. A member is, however, allowed to transfer his or her membership from one approved society to another, provided the consent of the society from which the transfer is made is obtained. Such consent must not unreasonably be withheld, and with such transfer the reserve value standing to the credit of the member, according to the scale to be prescribed by the Insurance Commissioners, will pass to the account with the society to which the transfer is made. Although a society cannot prevent its members transferring, there is no compulsion on another society to admit a person desiring to transfer. Thus it may well happen, in the case of an insured person who is a member of a small local society, that by removal from the district membership with that society will terminate, and if the insured person is in bad health he will find difficulty in gaining admittance to another society in his new locality. In the event of failure to gain admittance he will be compelled to join the deposit-insurance section, to his great disadvantage, as pointed out in our issue of last week. It would thus appear to be in the interests of insured persons that they should join approved societies whose operations extend throughout the country, and so obviate the difficulties and uncertainties of transfer.

This will be an important advantage of membership of the Nurses' National Insurance Society, quite apart from the special benefits which it will be possible for trained female nurses to obtain through the medium of the society.

Sickness Benefits, Reserves and Valuations.

In the same way that Life Assurance necessitates the accumulation out of the premiums paid of reserves to meet future claims when they arise, so also does sickness insurance require the accumulation of reserves. The adequacy of the reserves is as much a fundamental condition of the solvency of a society granting sickness benefits as it is of a Life Assurance Company, and the periodical valuation of the assets and liabilities of approved societies, on sound actuarial principles, thus becomes a necessity. The Act prescribes that all approved societies shall submit their affairs to valuation every three years, or at other intervals as may be required by the Insurance Commissioners. The valuer is to be appointed by or with the approval of the Treasury, and the valuation is to be made on such a basis as may be prescribed. It may be presumed that the valuation basis will be fixed by the Insurance Commissioners on national lines in the same way as the contributions and benefits have been determined at the outset. In other words, the valuation basis will probably be the same for all societies of whatever class they may be, though it cannot be asserted authoritatively that this will be so. Now, it is common knowledge that sickness rates vary over wide limits according to occupation, and that the rates of mortality, which also vary in different sections of the community, also have an important bearing on the value of sickness benefits.

A Surplus or Deficiency.

It is more than probable that no society will show an exact equality between the value of its assets on the one hand and of its liabilities on the other. In other words, the valuation will disclose either a surplus or a deficiency. The disclosure of a surplus will mean that the society has been so well administered, or that its experience has been so favourable, that it has funds in hand or to its credit in excess of its requirements on the prescribed basis. This surplus will thus be the result of the overpayment in contributions by the members for the standard benefits, and in equity it should be divided in an approved manner amongst the members of the society from whose over-payments it has arisen. On the other hand, if a deficiency is shown, it will indicate that the reserves in hand, though they may be considerable in actual amount, are not so large as they should be in order to meet the probable future claims as they emerge. The implication of such a state of affairs will be that the society has not been administered as well as it might have been, or that its experience is such that a higher contribution would have been justified. In equity this deficiency should be borne or made good by the individual society.

The Sinking of Small Societies.

In point of fact, however, an approved society which, at the date of any valuation, has less than 5,000 insured members under its State section will be joined with another society or other societies in an association, as it may elect, or according to the locality in which it carries on business, so that in the aggregate the number of insured members is not less than 5,000. Each society will then be separately valued, but if one disclose a surplus and another a deficiency, the society with a surplus will be liable to the extent of one-third of such surplus to contribute towards the liquidation of the deficiency on the associated society or societies. It will thus be seen that unless a minimum of 5,000 members is obtained the benefit of the favourable experience or good management, or both, of a society may not be fully realised by the members of such society, but may to the extent of one-third of the surplus be devoted to the benefit of others over whom the society has no control.

The Nurses' National Insurance Society.

This is a further reason why the female nursing staffs of the voluntary hospitals should support the Nurses' National Insurance Society by becoming members at the earliest possible opportunity, for there is little doubt that the society will experience a particularly favourable sickness-rate, and that it will be well managed goes without saying. Provided, therefore, that the necessary 5,000 members are obtained, of which surely there should be no doubt, the nurses would obtain through the society the full benefit of the surplus that may be expected to accrue. Various benefits alternative to those comprised in the standard schedule are allowed under the Act when the conditions of employment are such as to make the standard benefits unnecessary or inapplicable. We propose to deal with the benefits as suited to the requirements of nurses in our next issue.

